# Supplementary material for: Interpreter usage and associations with latent tuberculosis infection treatment acceptance and completion in the USA among non-U.S.–born persons, 2012–2017
Source: PLoS One. 2024 Apr 16;19(4):e0298628. doi: 10.1371/journal.pone.0298628 (PMC11020400; doi:10.1371/journal.pone.0298628)
Supplement: S1 Table — (DOCX) [file pone.0298628.s001.docx]

| **High risk countries** | | | |
| --- | --- | --- | --- |
| Afghanistan | Dominican Republic | Lesotho | Sao Tome and Principe |
| Angola | East Timor | Liberia | Senegal |
| Azerbaijan | Ecuador | Libya | Seychelles |
| Bangladesh | Equatorial Guinea | Madagascar | Sierra Leone |
| Benin | Eritrea | Malawi | Singapore |
| Bhutan | Ethiopia | Maldives | Solomon Islands |
| Bolivia | Federated States of Micronesia | Mali | Somalia |
| Botswana | Gabon | Marshall Islands | Sri Lanka |
| Brunei | Gambia | Mauritania | Swaziland |
| Burkina Faso | Georgia | Mauritius | Tanzania |
| Burundi | Guatemala | Mongolia | Tibet |
| Cambodia | Guinea | Mozambique | Togo |
| Cameroon | Guinea-Bissau | Myanmar (Burma) | Tunisia |
| Cape Verde | Haiti | Namibia | Tuvalu |
| Central African Republic | Honduras | Nepal | Uganda |
| Chad | India | Niger | Vietnam |
| China | Indonesia | Pakistan | Yemen |
| Comoros | Ivory Coast | Papua New Guinea | Zambia |
| Congo | Kenya | Peru | Zimbabwe |
| Democratic Republic of Congo | Kiribati | Philippines |  |
| Djibouti | Laos | Rwanda |  |
