# Supplementary material for: Interpreter usage and associations with latent tuberculosis infection treatment acceptance and completion in the USA among non-U.S.–born persons, 2012–2017
Source: PLoS One. 2024 Apr 16;19(4):e0298628. doi: 10.1371/journal.pone.0298628 (PMC11020400; doi:10.1371/journal.pone.0298628)
Supplement: S2 Table — (DOCX) [file pone.0298628.s002.docx]

|  | **Total^1^**  N_1_ = 8,761 | | **Interview in English**  N_2_ = 2,489 | | **Interview in language other than English**  N_3_ = 6,272 | |
| --- | --- | --- | --- | --- | --- | --- |
| Enrollment Sites | No. | % of N_1_ | No. | % of N_2_ | No. | % of N_3_ |
| Baltimore City (MD) Health Department | 659 | 7.52 | 122 | 4.9 | 537 | 8.6 |
| Atrium Health (Charlotte, NC) | 400 | 4.57 | 14 | 0.6 | 386 | 6.2 |
| DeKalb County (GA) Board of Health | 1,679 | 19.16 | 49 | 2.0 | 1,630 | 26.0 |
| Denver (CO) Public Health | 776 | 8.86 | 177 | 7.1 | 599 | 9.6 |
| Florida Department of Health/Broward County—Ft. Lauderdale | 354 | 4.04 | 177 | 7.1 | 177 | 2.8 |
| Public Health—Seattle (WA) and King County | 893 | 10.19 | 399 | 16.0 | 494 | 7.9 |
| Hawaii Department of Health | 810 | 9.25 | 645 | 25.9 | 165 | 2.6 |
| Maricopa County (AZ) Department of Public Health | 841 | 9.6 | 106 | 4.3 | 735 | 11.7 |
| Montgomery County (MD) Department of Health and Human Services | 29 | 0.33 | 20 | 0.8 | 9 | 0.1 |
| Florida Department of Health/Miami-Dade County—Miami | 28 | 0.32 | 12 | 0.5 | 16 | 0.3 |
| Metro Public Health Department—Nashville (TN) | 325 | 3.71 | 90 | 3.6 | 235 | 3.8 |
| Florida Department of Health/Alachua County—Gainesville | 122 | 1.39 | 114 | 4.6 | 8 | 0.1 |
| Florida Department of Health/Broward County—Pompano Beach | 187 | 2.13 | 83 | 3.3 | 104 | 1.7 |
| County of San Diego Health and Human Services Agency | 279 | 3.18 | 130 | 5.2 | 149 | 2.4 |
| San Francisco (CA) Department of Public Health | 582 | 6.64 | 223 | 9.0 | 359 | 5.7 |
| Tarrant County (TX) Health Department | 703 | 8.02 | 60 | 2.4 | 643 | 10.3 |
| University of California San Diego Antiviral Research Center UCSD | 21 | 0.24 | 8 | 0.3 | 13 | 0.2 |
| Wake County (NC) Health Department | 73 | 0.83 | 60 | 2.4 | 13 | 0.2 |

^1^The data for interpreter use for 16 enrolled participants were missing from the following sites: San Francisco Department of Public Health (1), Maricopa County Department of Public Health (14), Dekalb County Board of Health (1)
