# Supplementary material for: Interpreter usage and associations with latent tuberculosis infection treatment acceptance and completion in the USA among non-U.S.–born persons, 2012–2017
Source: PLoS One. 2024 Apr 16;19(4):e0298628. doi: 10.1371/journal.pone.0298628 (PMC11020400; doi:10.1371/journal.pone.0298628)
Supplement: S3 Table — (DOCX) [file pone.0298628.s003.docx]

| Characteristic | Total  **N = 6,272** | | Bilingual  **N = 2,151** | | In-person  **N = 2,248** | | Telephone  **N = 1,873** | |
| --- | --- | --- | --- | --- | --- | --- | --- | --- |
| Age in years | No. | % | No. | % | No. | % | No. | % |
| 0-14 | 806 | 12.9% | 335 | 15.6% | 265 | 11.8% | 206 | 11.0% |
| 15-24 | 966 | 15.4% | 329 | 15.3% | 367 | 16.3% | 270 | 14.4% |
| 25-44 | 2,923 | 46.5% | 1,022 | 47.5% | 1,117 | 49.7% | 784 | 41.9% |
| 45-64 | 1,289 | 20.6% | 393 | 18.3% | 402 | 17.9% | 494 | 26.4% |
| $\geq$65 | 288 | 4.6% | 72 | 3.4% | 97 | 4.3% | 119 | 6.4% |
| Gender |  |  |  |  |  |  |  |  |
| Women | 2,934 | 46.7% | 967 | 45.0% | 1,098 | 48.8% | 869 | 46.4% |
| Men | 3,336 | 53.3% | 1,183 | 55.0% | 1,149 | 51.1% | 1,004 | 53.6% |
| Transgender | 2 | 0.0% | 1 | 0.1% | 1 | 0.0% | 0 | 0.0% |
| Race/Ethnicity |  |  |  |  |  |  |  |  |
| Asian | 2,329 | 37.1% | 947 | 44.0% | 832 | 37.0% | 550 | 29.4% |
| Black/African American | 900 | 14.4% | 32 | 1.5% | 473 | 21.0% | 395 | 21.1% |
| Hispanic/Latino | 851 | 13.6% | 498 | 23.2% | 274 | 12.2% | 79 | 4.2% |
| White | 214 | 3.4% | 41 | 1.9% | 88 | 3.9% | 85 | 4.5% |
| Pacific Islander | 31 | 0.5% | 2 | 0.1% | 1 | 0.0% | 28 | 1.5% |
| Native American | 2 | 0.0% | 0 | 0.0% | 0 | 0.0% | 2 | 0.1% |
| Other | 1,565 | 25.0% | 510 | 23.7% | 425 | 18.9% | 630 | 33.6% |
| Unknown | 380 | 6.1% | 121 | 5.6% | 155 | 6.9% | 104 | 5.6% |
| Time since arrival to US |  |  |  |  |  |  |  |  |
| <5 years | 5,453 | 86.9% | 1,841 | 85.6% | 1,914 | 85.1% | 1,698 | 90.7% |
| ≥5 years | 819 | 13.1% | 310 | 14.4% | 334 | 14.9% | 175 | 9.3% |
| Enrollment reason |  |  |  |  |  |  |  |  |
| Close contact | 343 | 5.5% | 147 | 6.8% | 65 | 2.9% | 131 | 7.0% |
| Foreign born | 5,620 | 89.6% | 1,867 | 86.8% | 2,027 | 90.2% | 1,726 | 92.2% |
| Member of group with local LTBI prevalence $\geq$25%^1^ | 289 | 4.6% | 120 | 5.6% | 155 | 6.9% | 14 | 0.8% |
| Spent at least 30 days in a high-risk country^3^ in the last 5 years^2^ | 5 | 0.1% | 3 | 0.1% | 1 | 0.0% | 1 | 0.1% |
| HIV positive | 15 | 0.2% | 14 | 0.7% | 0 | 0.0% | 1 | 0.1% |
| Education |  |  |  |  |  |  |  |  |
| No schooling | 1,021 | 16.3% | 339 | 15.8% | 376 | 16.7% | 306 | 16.3% |
| Eighth grade or less | 2,491 | 39.7% | 905 | 42.1% | 893 | 39.7% | 693 | 37.0% |
| Some high school | 1,028 | 16.4% | 310 | 14.4% | 396 | 17.6% | 322 | 17.2% |
| High school graduate or GED | 1,037 | 16.5% | 393 | 18.3% | 324 | 14.4% | 320 | 17.1% |
| Trade school or associate degree | 60 | 1.0% | 15 | 0.7% | 16 | 0.7% | 29 | 1.6% |
| Some university/college | 222 | 3.5% | 67 | 3.1% | 83 | 3.7% | 72 | 3.8% |
| University/college graduate | 346 | 5.5% | 103 | 4.8% | 135 | 6.0% | 108 | 5.8% |
| Postgraduate schooling | 37 | 0.6% | 12 | 0.6% | 16 | 0.7% | 9 | 0.5% |
| Other | 7 | 0.1% | 1 | 0.1% | 0 | 0.0% | 6 | 0.3% |
| Don't know/refused | 23 | 0.4% | 6 | 0.3% | 9 | 0.4% | 8 | 0.4% |
| Region of birth country |  |  |  |  |  |  |  |  |
| Africa | 647 | 10.3% | 27 | 1.3% | 359 | 16.0% | 261 | 13.9% |
| America | 1,288 | 20.5% | 640 | 29.8% | 418 | 18.6% | 230 | 12.3% |
| Europe | 52 | 0.8% | 27 | 1.3% | 11 | 0.5% | 14 | 0.8% |
| Mediterranean | 857 | 13.7% | 3 | 0.1% | 403 | 17.9% | 451 | 24.0% |
| Pacific | 797 | 12.7% | 127 | 5.9% | 228 | 10.1% | 442 | 23.6% |
| Southeast Asia | 2,631 | 42.0% | 1,327 | 61.7% | 829 | 36.9% | 475 | 25.4% |
| Experiencing homelessness |  |  |  |  |  |  |  |  |
| Yes | 76 | 1.2% | 29 | 1.3% | 25 | 1.1% | 22 | 1.2% |
| Don’t know/refused | 13 | 0.2% | 0 | 0.0% | 3 | 0.1% | 10 | 0.5% |
| HIV infection |  |  |  |  |  |  |  |  |
| Yes | 49 | 0.8% | 27 | 1.3% | 7 | 0.3% | 15 | 0.8% |
| Don’t know/refused | 54 | 0.9% | 9 | 0.4% | 34 | 1.5% | 11 | 0.6% |
| Diabetes |  |  |  |  |  |  |  |  |
| Yes | 263 | 4.2% | 66 | 3.1% | 86 | 3.8% | 111 | 5.9% |
| Don’t know/refused | 47 | 0.8% | 6 | 0.3% | 21 | 0.9% | 20 | 1.1% |
| LTBI Treatment regimen offered |  |  |  |  |  |  |  |  |
| 6- or 9- months isoniazid | 468 | 7.5% | 138 | 6.4% | 123 | 5.5% | 207 | 1.1% |
| 4 months rifampin | 1,315 | 21.0% | 232 | 10.8% | 555 | 24.7% | 528 | 2.8% |
| 12 weeks- weekly doses isoniazid/ rifapentine | 719 | 11.5% | 52 | 2.4% | 500 | 22.2% | 167 | 0.9% |
| Other | 767 | 12.2% | 564 | 26.2% | 151 | 6.7% | 52 | 0.3% |
| LTBI Treatment regimen Accepted |  |  |  |  |  |  |  |  |
| 6- or 9-months isoniazid | 903 | 14.4% | 204 | 9.5% | 383 | 17.0% | 316 | 16.9% |
| 4 months rifampin | 1,624 | 25.9% | 640 | 29.8% | 637 | 28.3% | 347 | 18.5% |
| 12 weeks- weekly doses isoniazid/ rifapentine | 369 | 5.9% | 48 | 2.2% | 222 | 9.9% | 99 | 5.3% |
| Other regimens | 139 | 2.2% | 65 | 3.0% | 31 | 1.4% | 43 | 2.3% |
| Age at arrival to US |  |  |  |  |  |  |  |  |
| $<$15 years | 855 | 13.6% | 367 | 17.1% | 276 | 12.3% | 212 | 11.3% |
| $\geq$15 years | 5,417 | 86.4% | 1,784 | 82.9% | 1,972 | 87.7% | 1,661 | 88.7% |
| Treatment outcome |  |  |  |  |  |  |  |  |
| Completed LTBI treatment | 2,415 | 63.7% | 797 | 37.1% | 1,046 | 46.5% | 572 | 30.5% |
| Did not complete LTBI treatment | 616 | 16.3% | 160 | 7.4% | 222 | 9.9% | 234 | 12.5% |

^1^Populations with a prevalence of LTBI > 25% varied by site (e.g., individuals experiencing homelessness or have a specific medical condition)

^2^Refer to supplemental table 1 for a list of high-risk countries
