# Supplementary material for: Interpreter usage and associations with latent tuberculosis infection treatment acceptance and completion in the USA among non-U.S.–born persons, 2012–2017
Source: PLoS One. 2024 Apr 16;19(4):e0298628. doi: 10.1371/journal.pone.0298628 (PMC11020400; doi:10.1371/journal.pone.0298628)
Supplement: S4 Table — N = 8,402. (DOCX) [file pone.0298628.s004.docx]

| **Characteristics** | **Adjusted odds ratio** | **95% confidence -interval** | **p-value** |
| --- | --- | --- | --- |
| **Interpreter** | 0.98 | 0.82 – 1.16 | 0.80 |
| **Time in the US** | 0.98 | 0.97 – 0.99 | <0.001 |
| **Gender** |  |  |  |
| Women | reference |  |  |
| Men | 1.05 | 0.94 – 1.17 | 0.43 |
| Transgender | 0.63 | 0.05 – 8.57 | 0.73 |
| **Enrollment reason** |  |  |  |
| Close contact | reference |  |  |
| Non-U.S.–born | 0.29 | 0.23 – 0.37 | <0.001 |
| Member of group with local LTBI $\geq$25%^1^ | 0.25 | 0.17 – 0.39 | <0.001 |
| Spent at least 30 days in a high-risk country in the last 5 years^2^ | 0.34 | 0.10 – 1.18 | 0.09 |
| HIV infection | 0.29 | 0.09 – 0.90 | 0.03 |
| **Age** | 1.00 | 1.0 – 1.0 | 0.82 |
| **Race/ethnicity** |  |  |  |
| Asian | reference |  |  |
| Black/African American | 0.87 | 0.66 – 1.16 | 0.35 |
| Hispanic/Latino | 1.10 | 0.76 – 1.59 | 0.63 |
| White | 1.11 | 0.76 – 1.63 | 0.59 |
| Pacific Islander | 0.64 | 0.41 – 1.0 | 0.048 |
| Other | 0.94 | 0.79 – 1.12 | 0.52 |
| Unknown | 0.79 | 0.59 – 1.06 | 0.11 |
| **Region of birth country** |  |  |  |
| Africa | reference |  |  |
| America | 0.74 | 0.51 – 1.08 | 0.12 |
| Europe | 0.43 | 0.22 – 0.82 | 0.01 |
| Mediterranean | 0.99 | 0.76 – 1.28 | 0.92 |
| Pacific | 0.68 | 0.48 – 0.96 | 0.03 |
| Southeast Asia | 1.03 | 0.76 – 1.40 | 0.83 |
| **Education** |  |  |  |
| No schooling | reference |  |  |
| Eighth grade or less | 1.16 | 0.96 – 1.40 | 0.13 |
| Some high school | 1.19 | 0.96 – 1.49 | 0.12 |
| High school graduate or GED | 0.96 | 0.77 – 1.19 | 0.71 |
| Trade school or associates degree | 1.44 | 0.94 – 2.19 | 0.09 |
| Some university/college | 1.16 | 0.88 – 1.53 | 0.29 |
| University/college graduate | 1.07 | 0.83 – 1.38 | 0.59 |
| Postgraduate schooling | 0.72 | 0.48 – 1.10 | 0.13 |
| Other | 1.57 | 0.17 – 14.08 | 0.69 |
| Don't know/refused | 0.87 | 0.36 – 2.13 | 0.76 |
| **Experiencing homelessness** | 0.41 | 0.26 – 0.65 | <0.001 |
| **HIV** |  |  |  |
| HIV positive | 1.26 | 0.63 – 2.50 | 0.52 |
| **Diabetes** | 1.04 | 0.80 – 1.34 | 0.79 |
| **Tuberculin Skin Test** | 1.54 | 1.29 – 1.84 | <0.001 |
| **QuantiFERON TB Gold In-Tube** | 14.29 | 12.51 – 16.32 | <0.001 |

T-SPOT.TB test excluded due to >10% missing data

^1^Populations with a prevalence of LTBI > 25% varied by site (e.g., individuals experiencing homelessness or have a specific medical condition)

^2^Refer to supplemental table 1 for a list of high-risk countries
