# Supplementary material for: Interpreter usage and associations with latent tuberculosis infection treatment acceptance and completion in the USA among non-U.S.–born persons, 2012–2017
Source: PLoS One. 2024 Apr 16;19(4):e0298628. doi: 10.1371/journal.pone.0298628 (PMC11020400; doi:10.1371/journal.pone.0298628)
Supplement: S6 Table — N = 3,114. (DOCX) [file pone.0298628.s006.docx]

| **Characteristics** | **Adjusted odds ratio** | **95% confidence -interval** | **p-value** |
| --- | --- | --- | --- |
| **Interpreter type** |  |  |  |
| Telephone-based | reference |  |  |
| In-person | 0.87 | 0.51 – 1.49 | 0.62 |
| Bilingual study interviewer | 0.92 | 0.47 – 1.79 | 0.80 |

*Variables that were adjusted for included: time in the United States, gender, enrollment reason, age, race/ethnicity, region of birth, education, HIV infection, diabetes, experiencing homelessness, LTBI treatment regimen, TB infection test
